# Probe-based confocal laser endomicroscopy: progress, challenges, and emerging applications

**DOI:** 10.1007/s00464-025-12297-w

**Published:** 2025-11-03

**Authors:** Mohammed Ayyad, Dhir Gala, Maram Albandak, Ritik M. Goyal, Yazan Abboud, Ahmed Al-Khazraji, Kaveh Hajifathalian

**Affiliations:** 1 Department of Medicine, Rutgers New Jersey Medical School, 185 S Orange Ave, Newark, NJ 07103 USA; 2 Department of Medicine, University of Toledo, Toledo, OH USA; 3 UCLA Division of Digestive Diseases, University of California, Los Angeles, CA USA; 4 Division of Gastroenterology and Hepatology, Rutgers New Jersey Medical School, Newark, NJ USA

**Keywords:** Confocal endomicroscopy, pCLE, Gastrointestinal imaging, Barrett’s esophagus, Inflammatory bowel disease, Real-time biopsy

## Abstract

**Background & Aims:**

Probe-based confocal laser endomicroscopy (pCLE) provides real-time, cellular-level “optical biopsy” during endoscopy. This review synthesizes the technology, diagnostic performance, clinical use, safety, costs, and future directions of pCLE across gastrointestinal indications.

**Methods:**

Targeted narrative review of randomized trials, meta-analyses, and observational studies on pCLE in Barrett’s esophagus, gastric intestinal metaplasia (GIM), colorectal neoplasia, inflammatory bowel disease (IBD), and indeterminate biliary strictures. Outcomes included sensitivity/specificity, impact on biopsy yield and management, adverse events, and cost effectiveness.

**Results:**

pCLE combines a fiber-optic probe and IV fluorescein with 488-nm excitation to generate optical sections (~ 1 µm lateral resolution; depth ~ 55–70 µm). In Barrett’s esophagus, adding pCLE to high-definition endoscopy nearly doubled neoplasia detection sensitivity versus standard protocols. For GIM, pooled sensitivity and specificity reached 97% and 94%. For colorectal lesions, sensitivity 81–91% and specificity 75–91% allowed in vivo characterization and fewer unnecessary resections. In IBD surveillance, pCLE identified more neoplasia with fewer biopsies (up to ~ 4.7-fold increase) and pooled sensitivity/specificity of 87–100%/90–94%. In indeterminate biliary strictures, pCLE integrated with ERCP/EUS improved accuracy and achieved negative predictive values up to 100%. Safety is favorable; risks relate mainly to fluorescein. Limitations include superficial penetration, narrow field of view, operator dependence, image-interpretation variability, procedure time, and high capital and per-case costs. Current guidelines view pCLE as an adjunct rather than routine standard. Emerging AI-assisted interpretation and molecular/ multispectral probes may standardize reads, expand indications, and improve yield.

**Conclusions:**

pCLE strengthens endoscopic decision-making by enabling immediate, near-histologic assessment, targeted sampling, and earlier therapy. Broader adoption hinges on standardized training, validated image criteria, multicenter randomized trials with health-economic endpoints, and integration of AI and molecular imaging to reduce variability and cost.

Probe-based confocal laser endomicroscopy (pCLE) is an advanced endoscopy-based imaging technique that allows providers to view the histology of tissues in real time [1, 2]. Confocal endomicroscopy was first developed in the early 2000s and has since evolved from bulky dedicated endoscopes to slender probe-based systems that are now able to pass through standard endoscope channels [3]. By utilizing laser scanning microscopy through a miniaturized probe, providers are able to visualize cellular and subcellular structures, allowing them to diagnose lesions that traditionally require to be biopsied [4, 5]. The most significant advantage and the main driving force for pCLE’s development is its ability to diagnose and characterize tissues in real-time during endoscopy and reduce the need for biopsy [6–8]. This also allows clinicians to make treatment decisions immediately and decreases the need for biopsies and additional unnecessary procedures.

This paper provides an integrated perspective that highlights pCLE’s role in IBD surveillance, its cost-effectiveness compared with random biopsies, and its emerging applications beyond gastroenterology. By combining current evidence with future directions such as AI and molecular imaging, we offer a broader clinical framework for pCLE as both a diagnostic tool and a platform for precision endoscopy.

## Technology and principles of pCLE

### Technology

**Fig. 1:**
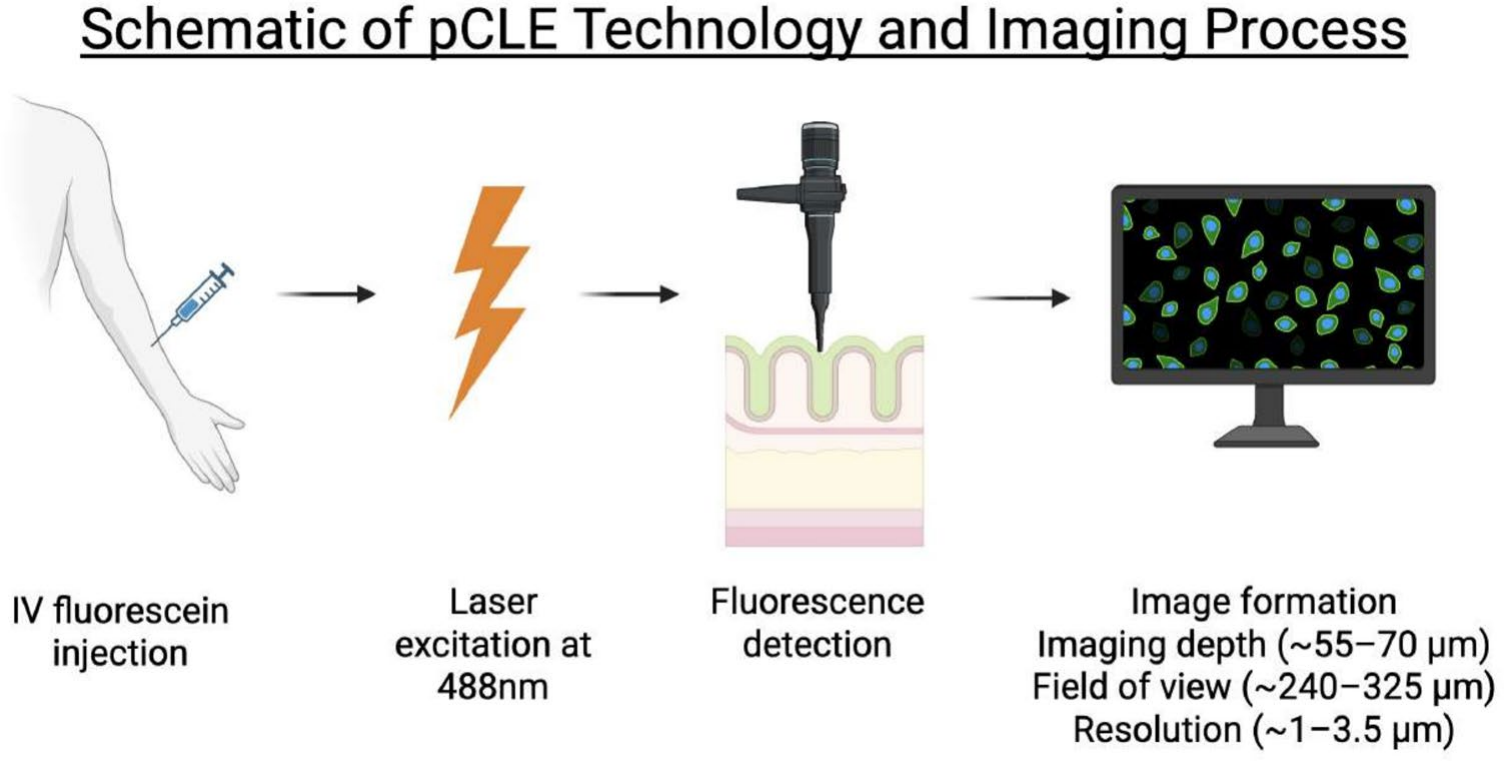
Schematic overview of the pCLE imaging process, highlighting fluorescein injection, laser excitation, fluorescence detection, and real-time image formation

pCLE uses an advanced and sophisticated approach to create high-resolution microscopic images of the mucosa. Figure 1 showcases a schematic overview of the pCLE technology and image formation. Initially, a fluorescent agent, most commonly intravenous sodium fluorescein, is administered. This quickly distributes through the vasculature and extravasates into the tissue matrix. A low-power 488 nm laser is then used for fluorescence excitation, and as the laser-scanned probe contacts the mucosal surface, emitted fluorescence is refocused through a pinhole, yielding optical section images ~ 1 μm in lateral resolution. It is able to create an image of approximately 55–70 μm from the surface, making it limited to the superficial mucosa [9–13].

This roughly corresponds to the epithelium and upper lamina propria of gastrointestinal (GI) organs, allowing for visualization of cellular morphology including glands, crypts, and capillaries, making it comparable to histology. This is supported by a prospective study by Bok et al. involving 54 lesions across 46 patients, where pCLE showed a higher level of agreement with final histopathology (*κ* = 0.824) compared to conventional endoscopic biopsies (*κ* = 0.617, *P* < 0.001). pCLE also demonstrated greater accuracy in detecting gastric adenocarcinoma (91.7%) versus standard biopsies (85.2%), and when both methods were combined, diagnostic accuracy increased to 98.1% [14]. Similarly, Vennelaganti et al. evaluated pCLE for identifying colon polyp types and found it could accurately distinguish hyperplastic polyps from tubular adenomas with an overall accuracy of 84.9% and substantial interobserver agreement (*κ* = 0.73), supporting its reliability across different users [15]. In another study, Li et al. used pCLE to assess disease activity in ulcerative colitis and found strong correlation with traditional histology, especially in detecting changes in crypt structure and blood vessels, reinforcing pCLE’s usefulness in evaluating inflammatory bowel disease in real time [16]. These findings are made possible by advancements in pCLE technologies, as dynamic scanning at 9–12 frames per second allows the endoscopist to create a real-time video and scan the probe across a mucosal area [7, 17]. However, due to the imaging limitation and a fixed focal plane, deeper structures present in the submucosa and beyond are not visible. Table 1 summarizes an overview of the pCLE technology and probe specifications.

**Table 1:** Overview of the technology, probe specifications, imaging characteristics, and interpretive frameworks for probe-based confocal laser endomicroscopy

Technology	–Intravenous fluorescein sodium is administered as a contrast agent –A 488 nm laser excites the fluorescein, and emitted fluorescence is refocused through a pinhole to form high-resolution optical sections (~ 1 μm lateral resolution) –Imaging depth: ~ 55 to 70 μm (limited to superficial mucosa) –Structures visualized: epithelium, upper lamina propria, glands, crypts, capillaries –Frame rate: 9–12 fps, enabling real-time video imaging
Imaging limitations	–Fixed focal plane –Submucosal and deeper structures are not visible –Visualization limited to surface mucosal architecture
Probe design	–Uses confocal mini-probes passed through the accessory channel of a standard endoscope –No dedicated endoscope required –Probes connect to a central LSU and digital display –Dual live monitors may assist in visualizing larger areas –Imaging performed continuously or in targeted snapshots post fluorescein injection
Common GI probes	GastroFlex™/ColoFlex™ Channel size: ≥ 2.8 mm Resolution: ~ 1.0 μm Field of View: ~ 240 μm CholangioFlex™ Channel size: ~ 1.0 mm Resolution: ~ 3.5 μm FOV: ~ 325 μm AQ-Flex™ (Needle-based CLE, nCLE) Used via EUS needle for in vivo pancreatic cyst imaging
Image interpretation	Real-time video sequences allow live interpretation by the endoscopist Recordings are digitally stored for frame-by-frame post-analysis Enables dynamic mucosal assessment like virtual histology
Classification systems	GI Lesions Mainz Classification and Miami Classification Features: epithelial thickness, glandular architecture, fluorescein leakage Barrett’s esophagus: dysplasia diagnosed by villiform irregularity, dark clumps Accuracy (trained users): sensitivity 88%, specificity 96%, κ ≈ 0.72 Biliary strictures Miami Classification: malignancy = thick white bands > 20 μm or dark cellular clumps Paris Classification: added benign features (e.g., vascular congestion, “scale-like” granular patterns) to improve specificity

*pCLE* probe-based confocal laser endomicroscopy, *GI* gastrointestinal, *fps* frames per second, *LSU* laser scanning unit, *FOV* field of view, *CLE* confocal laser endomicroscopy, *nCLE* needle-based confocal laser endomicroscopy, *EUS* endoscopic ultrasound

### Probe design and endoscope integration

Owing to the convenient design of the pCLE probe, this technology is considered an add-on rather than requiring a dedicated scope. pCLE uses flexible fiber-optic probes called confocal mini-probes that can be passed through an accessory channel in a standard endoscope. The endoscopist can choose from a variety of differently designed scopes tailored to different anatomical targets. GastroFlex and ColoFlex are GI confocal endomicroscopy probes that require a ≥ 2.8 mm channel and offer ultra-high definition imaging with ~ 1.0 μm resolution and a ~ 240 μm field of view [9, 18]. On the other hand, thinner probes like the CholangioFlex are used for biliary and pancreatic ducts. These probes can fit through a ~ 1.0 mm channel, providing a wider ~ 325 μm field of view at ~ 3.5 μm resolution [19–21]. Additionally, the AQ-Flex probe enables in vivo imaging inside pancreatic cysts via EUS-guided needle-based CLE (nCLE) [22, 23]. All the probes connect to the central laser scanning unit and display, allowing for seamless switching during a procedure. Some systems also contain dual live monitors to compensate for the small field of view and allow the endoscopist to examine multiple areas of interest by moving the probe tip. It is to be noted that pCLE imaging does not substantially alter endoscopic technique. The probe is simply applied to the anatomical target after a fluorescein injection, and the mucosa can be imaged continuously or in targeted snaps. The device also digitally records the sequences to allow clinicians to review them frame by frame at a later period.

### Image acquisition and interpretation

pCLE allows endoscopists to interpret microscopic features in real time by generating a video sequence of microscopic mucosal views. One major focus has been the standardization of image interpretations, due to which several classification systems have been developed to allow clinicians to translate confocal images into diagnostic criteria. Mainz classification and a parallel Miami classification have been the earliest classification criteria for GI lesions [24–26]. Both allow clinicians to classify tissue as neoplastic or non-neoplastic depending on features like epithelial thickness, glandular appearance, and fluorescein leakage. For instance, in Barrett’s esophagus, irregular villiform architecture, disorganized cells, and dark clumps check off the criteria for diagnosing that as dysplasia [27]. A published training study of pCLE in Barrett’s esophagus noted that endoscopists were able to identify neoplasia with 88% sensitivity and 96% specificity after getting taught a set of images, achieving substantial interobserver agreement (*κ* ≈ 0.72) [28]. The biliary tract has its own set of classification systems for strictures. The Miami classification defined malignancy as thick white bands > 20 μm or dark cellular clumps that yielded high sensitivity but moderate specificity. The Paris classification then introduced additional benign features to the classification criteria such as vascular congestion and dark granular “scale-like” patterns in order to reduce false positives caused by inflammatory strictures [29, 30].

## Clinical applications of pCLE

### Gastrointestinal applications

#### Esophagus

pCLE has been most extensively applied in Barrett’s esophagus with the aim of detecting dysplasia or early adenocarcinoma within Barrett’s mucosa during a surveillance endoscopy. A randomized controlled trial was conducted by Canto et al. in patients with Barrett’s esophagus, comparing standard HD white-light endoscopy (HD-WLE) with random biopsies versus HD-WLE plus pCLE targeted biopsies. pCLE allowed endoscopists to visually flag areas that raised suspicion and take targeted biopsies. The trial reported that use of pCLE nearly doubled the sensitivity for detecting BE-related neoplasia, from ~ 34% with standard endoscopy up to ~ 68% when pCLE was incorporated (per-biopsy analysis, *P* < 0.01). Clinically, this suggests that pCLE is capable of identifying dysplastic mucosal areas that random biopsies might overlook, improving the diagnostic yield of the endoscopy, and allowing for earlier therapy to be considered in these patients such as endoscopic mucosal resection (EMR) or radiofrequency ablation [31]. In one series, it was noted that pCLE assessment changed treatment plans of 36% of patients with Barrett’s esophagus, allowing for resection of an area that initially appeared normal under white light but was found to be suspicious under pCLE [31]. A prospective randomized crossover study, done by [32], showed that there is no difference in outcomes regarding the clinical impact of pCLE on patients with Barrett’s esophagus-related neoplasia, as AFI-guided pCLE had similar sensitivity for dysplasia compared with standard endoscopy using the Seattle protocol (*P* = 0.48) [32]. Due to continuously emerging evidence of pCLE’s role in early detection, consensus societies have recognized pCLE as a useful adjunct in Barrett’s esophagus, especially in patients who have a prior history of dysplasia. However, the ASGE’s guidelines from 2019 recommend against the routine use of CLE in BE surveillance, endorsing only a conditional recommendation due to low-quality evidence [33, 34]. The rationale behind this is that while pCLE increases dysplasia yield, the incremental benefit may not necessarily justify widespread use in routine surveillance given the costs and expertise needed for it. Table 2 summarizes the clinical applications and outcomes of pCLE.

**Table 2:** Summary of clinical appli﻿cation and outcomes evaluating probe-based confocal laser endomicroscopy (pCLE) across the field of endoscopic gastroenterology

GI region/condition	Key studies/references	Main findings	Sensitivity/specificity	Clinical impact
Esophagus (Barrett’s esophagus)	• Canto et al. (RCT) [31] • Society guidelines [30, 31, 34]	• pCLE nearly doubled the sensitivity for detecting BE-related neoplasia (from ~ 34 to ~ 68%) • pCLE altered treatment plans in ~ 36% of patients [31]	• Sensitivity improved from ~ 34 to ~ 68%	• Identifies dysplastic areas missed by random biopsies • Enables earlier endoscopic therapies (e.g., EMR, RFA) • Recognized as useful adjunct by some societies, but not fully endorsed for routine surveillance
Stomach (gastric intestinal metaplasia)	• Sun et al. (meta-analysis) [35]	• pCLE reliably identifies goblet cells and absorptive epithelium indicative of GIM • Allows real-time near-histological “optical biopsy”	• Pooled sensitivity: 97% • Specificity: 94%	• Enhances diagnostic yield by guiding targeted biopsies • Aids in detecting dysplasia for EMR/ESD • Improves surveillance efficiency
Colon (Polyps & IBD surveillance)	• Buchner et al. [36] • Kiesslich et al. [38]	• pCLE demonstrated higher sensitivity than NBI (91% vs. 77%) for differentiating neoplastic from non-neoplastic polyps • 4.75-fold more neoplasms detected in IBD surveillance	• Sensitivity: up to 91% (neoplastic vs. non-neoplastic) [36]	• Enables real-time polyp characterization, potentially avoiding unnecessary resections • Enhances IBD surveillance, detecting more neoplastic lesions with fewer biopsies
Biliary tract (indeterminate strictures)	• EMID study [44]	• pCLE via CholangioFlex probe during ERCP improved diagnostic accuracy and achieved 100% NPV when combined with endobiliary/EUS biopsies	• High sensitivity for malignancy; NPV: 100%	• Allows immediate visualization of stricture lining • Enhances preoperative assessment and clinical decision-making for biliary strictures
General GI tumor detection (various)	• Multiple references [2, 8, 39, 46–49, 82]	• pCLE yields higher sensitivity and specificity for GI malignancies compared to white-light endoscopy • Reduces unnecessary resections and repeat biopsies	• Meta-analysis of 9 studies [39] • Sensitivity: 87% • Specificity: 94% • AUROC: 0.96	• Minimizes risks and costs by targeted sampling • Improves clinical decision-making across different GI diseases
PSC-IBD-associated CRC surveillance	• Dlugosz et al. [80] • van den Broek et al. [81]	• Swedish study: Accuracy 96%, Sensitivity 89%, Specificity 96%, PPV 41%, NPV 99% • Lower diagnostic yield in inflamed areas • van den Broek study showed lower performance in early research	• Accuracy ~ 96%; high NPV (99%)	• Reliable for ruling out dysplasia in PSC-IBD • Operator experience is critical • Improved technology and methods over time
UC surveillance (neoplasia detection)	• Randomized study in 161 UC patients [38] • Rispo et al. [51]	• ~ 5 × increase in neoplasia detection with pCLE compared to standard colonoscopy • Sensitivity 100%, Specificity 90% for detecting dysplasia in UC	• Accuracy 97.8% in predicting neoplastic changes [38]	• Allows earlier intervention by identifying dysplasia in UC • Operator-dependent; training needed • Cost and procedure time are concerns
Pancreaticobiliary strictures (IPSs)	Multiple studies [74]	• pCLE significantly increases diagnostic sensitivity for early malignancy, preventing delayed diagnoses	• Enhances sensitivity over traditional ERCP sampling	• Reduces need for repeat procedures and associated risks • Improves timeliness of potential surgical intervention

*pCLE* Probe-based confocal laser endomicroscopy, *BE* Barrett’s esophagus, *EMR* Endoscopic mucosal resection, *RFA* Radiofrequency ablation, *GIM*: Gastric intestinal metaplasia, *ESD* Endoscopic submucosal dissection, *IBD* Inflammatory bowel disease, *NBI* Narrow-band imaging, *ERCP* Endoscopic retrograde cholangiopancreatography, *NPV* Negative predictive value, *AUROC* Area under the receiver operating characteristic curve, *PSC* Primary sclerosing cholangitis, *CRC* Colorectal cancer, *UC* Ulcerative colitis

#### Stomach

In the stomach, pCLE has shown promising advancement in the detection of precancerous and early cancerous changes, including gastric intestinal metaplasia (GIM) and dysplasia. Due to being a precursor to intestinal type gastric adenocarcinoma, gastric intestinal metaplasia often warrants extensive random biopsies to map its presence. pCLE allows the endoscopist to identify areas with intestinal metaplasia in real time by recognizing the characteristic appearance of goblet cells and absorptive epithelium within the gastric mucosa. A 2016 meta-analysis by Sun et al. combined 4 studies of 579 unspecified gastric lesions and found a pooled sensitivity of 97% and specificity of 94% for pCLE diagnosis of gastric intestinal metaplasia, supporting a low false-positivity rate [35]. A randomized controlled trial by Zuo et al. demonstrated that pCLE targeted biopsies significantly reduce the number of biopsies per patient by 48.5% as compared to flexible spectral imaging color enhancement (FICE) with standard biopsies (*P* < 0.001) [35]. pCLE allows for near-histological optical biopsy of the stomach, allowing targeted biopsies for intestinal metaplasia during surveillance endoscopy. This improves efficiency and diagnostic yield, while also aiding in delineating intestinal metaplasia or dysplasia for endoscopic resection. By detecting early gastric cancer features, pCLE helps facilitate real-time EMR or endoscopic submucosal dissection (ESD).

#### Colon

In the colon, pCLE is primarily used to characterize polyps during a colonoscopy. Specifically, it allows for distinguishing between adenomatous or neoplastic polyps from non-neoplastic polyps such as hyperplastic or inflammatory polyps in real time. A crucial study by Buchner et al. compared pCLE to virtual chromoendoscopy (Flexible spectral imaging color enhancement (FICE) or narrow-band imaging (NBI)) in classifying 119 colorectal polyps in vivo. In this study, the endoscopist performed pCLE after NBI inspection of a polyp, before resecting it for histology. It concluded that pCLE had significantly higher sensitivity than NBI for differentiating neoplastic vs. non-neoplastic polyps (91% vs. 77% sensitivity, *P* = 0.01) with similar specificity. The authors of the study suggested that pCLE can possibly replace histopathology, allowing for a real-time assessment of small polyps [36]. In a meta-analysis conducted by Dong et al., the sensitivity and specificity of pCLE in detecting colorectal neoplasia were 81% and 75%, respectively, hence a false-positive rate of 25% [37]. However, when image quality is high and interpretation is performed by experienced users, specificity improves, and false-positive rates decrease substantially [15].

Surveillance of the colon is also needed in longstanding ulcerative colitis (UC) or Crohn’s colitis to scan for dysplasia. pCLE has been explored in this field as well, to inspect colonic mucosa for dysplasia during IBD surveillance, yielding some promising early results. A study by Kiesslich et al. identified 4.75-fold more neoplasms with 50% fewer biopsies when pCLE was utilized compared to a traditional colonoscopy. It enabled real-time in vivo histologic assessment with high accuracy, potentially enhancing surveillance efficiency and clinical management [38]. A meta-analysis of nine studies found that pCLE demonstrated a pooled sensitivity of 87%, specificity of 94%, and an area under the receiver operating characteristic curve of 0.96 in distinguishing neoplastic from non-neoplastic colonic lesions in IBD patients [39]. In Crohn’s disease, the clinical yield of pCLE is less well established due to the lower incidence of dysplasia. Sensitivity for dysplasia detection may be as low as 42.9%, with specificity up to 92.4%, and overall accuracy of 86.7% [40]. The ASGE notes that while advanced imaging modalities like pCLE can improve lesion characterization, their use is largely restricted to expert centers and is not yet standard of care for routine surveillance in IBD. Chromoendoscopy remains the recommended technique for dysplasia detection, with pCLE considered an adjunct in select cases [41].

#### Biliary tract

One of the most fascinating uses of pCLE is in the evaluation of indeterminate biliary strictures. Endoscopists can use a small CholangioFlex probe which can be inserted through an ERCP catheter or cholangioscope into the bile duct. This allows for visualization and imaging of the stricture lining. The use of pCLE in imaging biliary strictures has been appraised in large part due to its high sensitivity for malignancy. However, false-positivity rates with pCLE are higher than with tissue sampling, primarily due to misclassification of benign inflammatory strictures as malignant. Early classification systems (Miami Criteria) yielded specificities as low as 67–71%, with false positives often related to inflammation or prior stenting. The development of the Paris Classification, which incorporates criteria for benign inflammatory changes, has improved specificity and reduced false positives, especially in patients with prior biliary stenting, raising specificity to 73–88% and overall accuracy to 71–79% [42, 43]. The EMID study has demonstrated that performing pCLE in addition to endobiliary and endoscopic ultrasound (EUS)-guided biopsies significantly improved the diagnostic accuracy in cases of biliary strictures. It conjunctively achieved a 100% negative predictive value and enhanced the preoperative histologic assessment [44]. The ACG notes that pCLE can help distinguish inflammatory from malignant strictures, but its performance characteristics do not yet meet the threshold for definitive oncologic decision-making at most institutions, and it remains an adjunct rather than a replacement for histopathology [45].

**Fig. 2:**
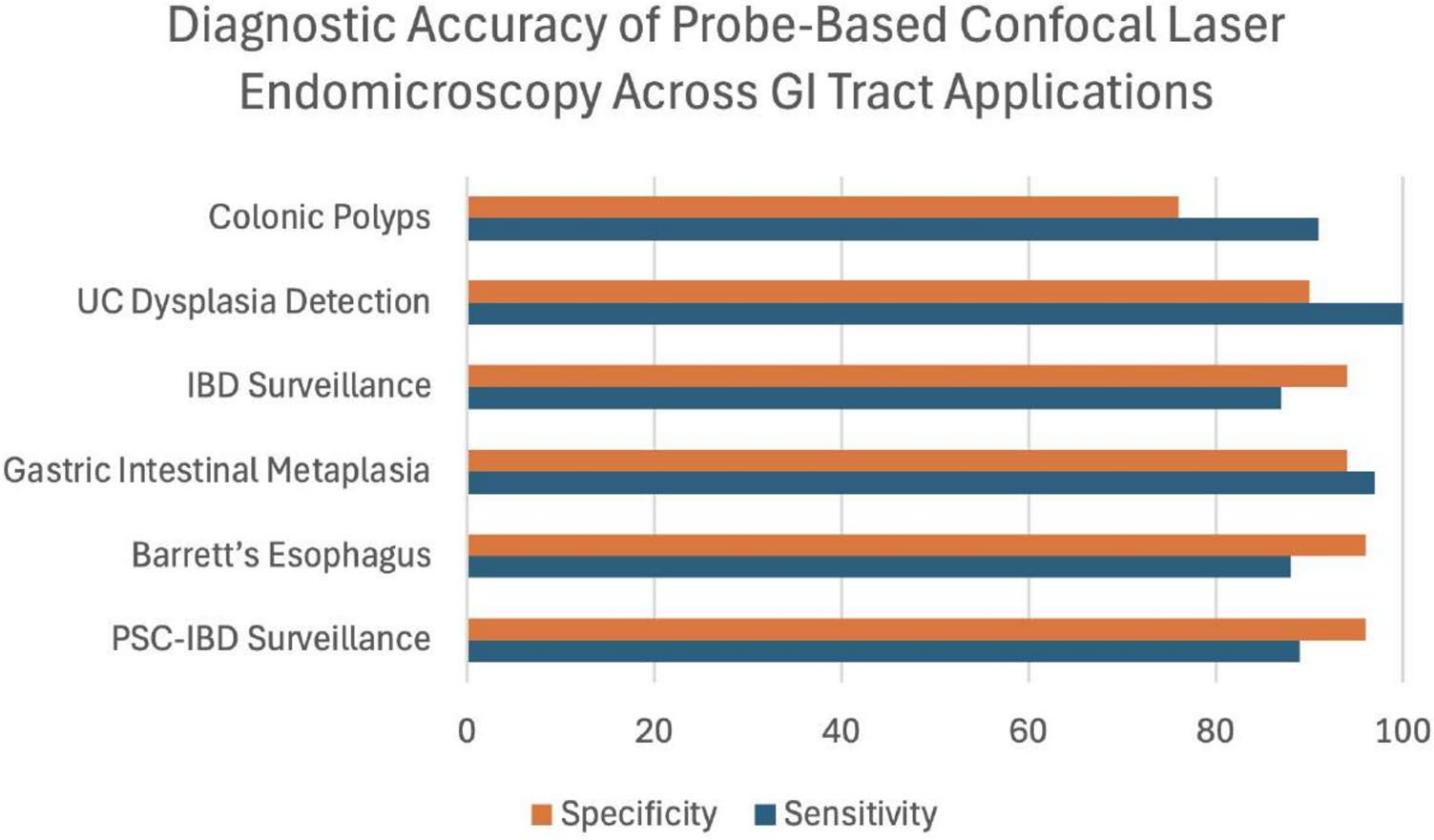
Diagnostic accuracy of pCLE showing sensitivity and specificity across various gastrointestinal tract applications

Overall, confocal laser endomicroscopy plays a crucial role in enhancing endoscopic diagnostics by minimizing unnecessary resections, reducing the need for repeated biopsies, and limiting unnecessary follow-up procedures. This, in turn, helps lower both the risks and costs associated with indiscriminate endoscopic examinations. Figure 2 showcases the diagnostic accuracy of pCLE across various GI applications.

**Fig. 3:**
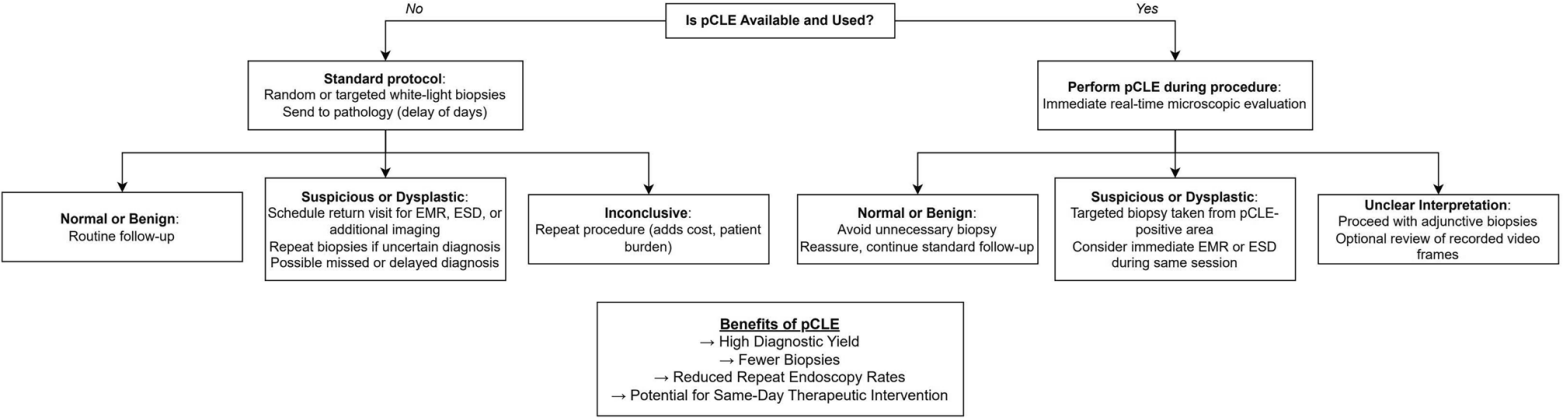
Decision algorithm illustrating the impact of pCLE use versus standard biopsy protocols on diagnostic efficiency and patient management in endoscopy

The primary clinical advantages of pCLE include real-time histopathological assessment, enhanced diagnostic accuracy, and improved clinical decision-making. Notably, pCLE has demonstrated higher sensitivity and specificity than conventional white-light endoscopy (WLE) for diagnosing gastrointestinal malignancies such as esophageal, gastric, and colorectal cancers [46, 47]. Additionally, pCLE facilitates targeted endoscopic biopsies, reducing the number of samples required for histopathological confirmation, while improving both the content and purity of malignant cells within biopsy specimens [48, 49]. In prior research, pCLE-guided biopsies yielded a significantly higher proportion of cancerous cells compared to conventional endoscopic biopsy techniques. This advantage was particularly evident in cases of gastric cancer with undifferentiated histology, where intraepithelial cancer cell content is typically low [49]. Table 3 highlights diagnostic performance and clinical recommendations for confocal laser endomicroscopy (CLE) across gastrointestinal indications, while Fig. 3 provides a suggested decision algorithm illustrating the impact of pCLE use versus standard biopsy protocols.

**Table 3:** Diagnostic performance and clinical recommendations for confocal laser endomicroscopy (CLE) across gastrointestinal indications

Indication	Sensitivity (%)	Specificity	False-positive rate	Clinical recommendations
Barrett’s esophagus	68	Variable	Not quantified, but risk of false-positive dysplasia calls exists	Useful adjunct in patients with prior dysplasia; ASGE 2019 *conditionally recommends against* routine use due to cost/low-quality evidence
Gastric intestinal metaplasia (GIM)/Dysplasia	97	94%	~ 6%	Reduces biopsy burden (~ 48% fewer per patient); aids in mapping GIM and detecting early gastric cancer; facilitates EMR/ESD
Colorectal polyps (general surveillance)	81–91	75–91%	Up to 25%	Effective for in vivo neoplasia characterization; can reduce unnecessary resections; adjunct to NBI/FICE, but not yet a replacement for histopathology
IBD surveillance (UC/Crohn’s colitis)	87–100	90–94%	6–10%	Detects more neoplasia with fewer biopsies; useful adjunct in PSC-IBD; operator-dependent; not standard of care—chromoendoscopy remains guideline-recommended
Biliary strictures (indeterminate/ERCP setting)	88–98	73–88%	High with inflammation/stents (misclassified as malignant)	Useful adjunct to ERCP + EUS biopsies; increases NPV (up to 100% in EMID study); ACG notes it’s not yet definitive for oncologic decisions

*ASGE* American Society for Gastrointestinal Endoscopy, *GIM* Gastric intestinal metaplasia, *EMR* Endoscopic mucosal resection, *ESD* Endoscopic submucosal dissection, *NBI* Narrow-band imaging, *FICE* Fuji intelligent color enhancement, *IBD* Inflammatory bowel disease, *UC* Ulcerative colitis. *PSC* Primary sclerosing cholangitis, *ERCP* Endoscopic retrograde cholangiopancreatography, *EUS* Endoscopic ultrasound, *NPV* Negative predictive value, *ACG* American college of gastroenterology, *EMID* Endomicroscopy in indeterminate biliary strictures

While multiple studies demonstrate high sensitivity and specificity for pCLE, many are limited by small sample sizes, single-center designs, and potential operator bias. Randomized controlled trials exist (e.g., Canto et al. in Barrett’s esophagus), but most available data are derived from observational cohorts, which may overestimate diagnostic yield [50]. Additionally, heterogeneity in classification systems (e.g., Miami vs. Paris criteria) introduces variability and complicates cross-study comparisons [29]. Few studies report blinding of endoscopists or pathologists, increasing the risk of interpretation bias. Follow-up periods are often short, limiting assessment of long-term outcomes such as recurrence or survival. Taken together, these methodological limitations reduce the certainty of current estimates and highlight the need for large, multicenter, blinded trials with standardized protocols to validate the routine use of pCLE.

## Cost-effectiveness of probe-based confocal laser endomicroscopy

In the context of UC, prior studies demonstrated that up to 33 random biopsies are required to achieve ~ 90% sensitivity for dysplasia detection, underscoring the need for more efficient, targeted imaging modalities like pCLE [51, 52]. In contrast, pCLE enables real-time, unlimited mucosal sampling, offering a more efficient and targeted approach to dysplasia detection.

Current literature on the cost-effectiveness of pCLE remains limited. A study presented at Digestive Disease Week 2016 evaluated its role in assessing indeterminate pancreaticobiliary strictures (IPSs) and found that incorporating pCLE into ERCP was more cost-effective than standard ERCP alone [53], demonstrating cost savings at a willingness-to-pay threshold of $50,000. These findings suggest that pCLE may enhance diagnostic accuracy while reducing the need for repeat procedures and associated healthcare costs. However, comprehensive cost-effectiveness analyses remain scarce, and further research is needed to compare its financial impact with standard diagnostic modalities.

## Complications and safety profile of pCLE

In terms of safety and adverse event rates, the American Society for Gastrointestinal Endoscopy (ASGE) and the Society of American Gastrointestinal and Endoscopic Surgeons (SAGES) Technology and Value Assessment Committee (TAVAC) have confirmed that pCLE possesses an excellent safety profile. The primary risks are related to the intravenous administration of fluorescein, rather than the procedure itself [54]. In a cross-sectional survey by Wallace et al. involving 2272 gastrointestinal pCLE procedures, no serious adverse events were observed. Mild adverse events occurred in 1.4% of cases and included symptoms such as nausea and vomiting, transient hypotension without shock, injection site erythema, diffuse rash, and mild epigastric discomfort [55].

Although pCLE is generally considered safe, some adverse events related to the procedure itself have been reported, most notably, pancreatitis, particularly in the context of biliary and pancreatic applications. Studies reporting the incidence of pancreatitis following nCLE include the INSPECT study, which noted a rate of 3.0%, and the DETECT study, which reported a higher rate of 6.7%. Factors contributing to the increased risk of pancreatitis with nCLE, as compared to standard EUS-FNA, include the use of larger 19-gauge needles, extended inspection times to visualize the cyst wall, and multi-directional needle manipulation within the cyst [22, 56]. Additionally, intra-cystic bleeding has been reported as a complication in approximately 5.5% of nCLE procedures involving pancreatic cysts [56]. Current recommendations include the administration of prophylactic antibiotics during and after the procedure, along with restricting the inspection time to under 10 min [54].

## Challenges in pCLE use: patient contraindications and procedural limitations

### Contraindications

Contraindications for pCLE are primarily associated with the use of intravenous fluorescein and the patient’s overall ability to tolerate endoscopic procedures. A known allergy to fluorescein is an absolute contraindication, as it poses a significant risk for hypersensitivity reactions. Similarly, fluorescein use is not recommended during pregnancy and lactation due to potential risks to the developing fetus or infants, although there is a lack of safety data on the use of fluorescein in pregnant or lactating women. Patients with severe renal impairment may also be at increased risk, as impaired clearance of fluorescein could lead to toxic accumulation. Additionally, individuals with high-risk cardiovascular conditions may not tolerate the transient hypotension that can occasionally follow fluorescein administration. Lastly, patients who are unable to safely undergo standard endoscopy due to severe comorbidities or poor general health are generally considered unsuitable candidates for pCLE [54–56].

### Limitations

While pCLE offers valuable real-time imaging capabilities, several limitations must be considered (Table 4). One major constraint is its limited penetration depth, typically reaching only the superficial mucosal layers (up to approximately 250 µm), which restricts its ability to evaluate deeper tissue structures. Additionally, the small field of view—just a few hundred micrometers—can hinder comprehensive assessment of larger lesions [57, 58]. Image quality is another challenge, as consistently obtaining high-resolution images requires significant technical expertise, and suboptimal image quality has been reported in a substantial number of pCLE recordings, potentially compromising diagnostic accuracy [59]. Furthermore, the interpretation of pCLE images is subject to interobserver variability, with differences often noted between endoscopists and pathologists, which can affect diagnostic consistency [60, 61]. The technique also demands specialized training, and the steep learning curve associated with both image acquisition and interpretation may limit its broader adoption [62]. Finally, the high cost of pCLE equipment, coupled with limited reimbursement options, poses significant barriers to routine clinical use [63]. These factors emphasize the importance of continued training, standardization, and validation to optimize the clinical utility of pCLE.

**Table 4:** Key limitations of probe-based confocal laser endomicroscopy

Limitation	Core issue	Clinical impact
Limited penetration depth	Confocal imaging reaches only ~ 250 µm into the superficial mucosa	Deeper layers remain unassessed, potentially missing important subsurface pathology
Small field of view	Imaging window spans a few hundred micrometers	Larger lesions may not be comprehensively visualized or evaluated
Image quality variability	Consistent high-resolution imaging requires refined technical expertise	Poor-quality images reduce diagnostic accuracy and demand additional training and hands-on experience
Interobserver variability	Differences in image interpretation between endoscopists and pathologists	Diagnostic consistency varies, mandating standardized interpretation protocols and ongoing training
Training and learning curve	Specialized training in both image acquisition and reading is required	Steep learning curve can delay adoption in routine practice, limiting broader clinical availability
High cost and reimbursement issues	Expensive equipment and limited financial coverage	Widespread deployment is restricted, particularly in resource-limited settings, inhibiting integration into standard practice

Currently, there is considerable variability in how clinicians are trained to perform and interpret pCLE, and the absence of standardized training and interpretation guidelines affects diagnostic consistency. Developing structured training curricula, certification programs, and centralized image libraries could help address this gap. Additionally, the lack of standardized interpretation protocols introduces subjectivity, and the integration of artificial intelligence (AI) tools may provide real-time decision support to reduce diagnostic variability and enhance reliability [3]. The technique is also highly operator-dependent, making outcomes variable across institutions and reinforcing the need for competency-based training. Moreover, economic and logistical considerations remain significant barriers—pCLE systems are expensive, procedure times are longer, and reimbursement is limited. While observational studies highlight the diagnostic accuracy and cost-saving potential of pCLE, large-scale randomized controlled trials comparing it with standard diagnostic pathways are still lacking. These limitations emphasize the importance of continued training, standardization, technological refinement, and validation to optimize the clinical utility of pCLE [3].

## Future directions

Although pCLE has been demonstrated to be highly diagnostically accurate in expert hands, its broader adoption into routine clinical practice is limited by several specific evidence shortcomings. Firstly, there is a lack of multicenter randomized controlled trials (RCTs) or head-to-head studies comparing pCLE-based versus traditional diagnostic algorithms for high-benefit indications such as surveillance of Barrett’s esophagus, the detection of inflammatory bowel disease, and the assessment of indeterminate biliary stricture. Most available evidence is the result of single-center studies in tertiary referral centers, and generalizability is therefore challenging. Future studies should be designed as multicenter RCTs that include both community and academic centers, with primary outcomes including the rate of neoplasia detection, negative predictive value, procedure time, and number of biopsies avoided, alongside secondary outcomes such as complication rate, patient-reported outcome, and cost-effectiveness.

Interpretation inconsistency remains another major hurdle, as current pCLE classification systems (Miami, Paris) have not been extensively validated across all clinical indications or practice settings. One significant area of advancement is the incorporation of artificial intelligence (AI) for real-time image analysis. Machine learning algorithms trained on large datasets of pCLE images have demonstrated the potential to automate and standardize image interpretation, reducing interobserver variability and improving diagnostic accuracy [64]. AI-assisted interpretation may allow for instant, objective identification of microscopic changes, thereby facilitating clinical decision-making during procedures without the need for extensive operator expertise [64, 65]. Preliminary studies have shown that deep learning models can match or exceed expert-level accuracy in distinguishing benign from malignant tissues, and future systems could provide real-time feedback during endoscopy, effectively functioning as a “digital pathologist” [66]. However, barriers to applying AI in pCLE include data heterogeneity, limited availability of large annotated datasets, operator dependency and interobserver variability in imaging protocols and devices, and the need for robust multicenter validation to ensure generalizability across populations and disease presentations [67–69]. Moreover, regulatory hurdles and validation requirements for AI in pCLE involve demonstration of safety, efficacy, and clinical utility through prospective, multicenter trials, as well as compliance with evolving regulatory frameworks such as those set by the U.S. Food and Drug Administration (FDA). The FDA requires rigorous validation, including reproducibility, transparency of algorithms, and post-market surveillance for AI-based medical devices [70].

No prospective studies, however, have evaluated AI-assisted pCLE in real-time clinical workflows. A research priority is the development of deep learning algorithms from large, annotated, multicenter image datasets and their validation in real-time clinical practice. The study design would be a prospective, crossover study in which operators interpret pCLE images with and without AI assistance and compare diagnostic accuracy, interobserver agreement, and decision-making time.

Parallel to AI integration, advancements in multi-spectral and molecular imaging hold promise to further enhance the diagnostic power of pCLE. Multi-spectral imaging, by enabling visualization of tissues across different light wavelengths, can highlight subtle biochemical and structural differences not visible under standard fluorescence [71]. Molecular imaging, using targeted fluorescent markers specific to tumor or inflammatory markers, could allow for selective enhancement of pathologic tissues [72, 73]. These innovations would extend the utility of pCLE beyond morphological assessment, enabling functional and molecular-level diagnostics during endoscopy. Targeted molecular probes, for instance against EGFR binding in dysplasia, VEGF for tumor angiogenesis, or immune cell markers for activity in IBD, would allow targeted visualization of high-risk lesions. Preclinical studies should be followed by initial first-in-human safety and pharmacokinetic trials, followed by prospective diagnostic accuracy studies in the same patient population comparing targeted pCLE to standard fluorescein-based pCLE. The primary outcome would be the incremental sensitivity gain for lesion detection, and secondary outcomes would assess the change in immediate therapeutic decision-making.

Even though operator dependence is recognized as a pCLE limitation, there are published data with quantitative estimates of the learning curve and training for proficiency. In a multicenter study by Buchner et al., novice endoscopists achieved diagnostic accuracy of approximately 65% for colorectal neoplasia after reading 20–30 cases, increasing to over 85% after review and execution of 50–75 cases [62]. These findings emphasize that pCLE adoption involves not only initial training but also ongoing case volume to maintain diagnostic performance, a factor that can render feasibility in low-volume institutions difficult.

The economic constraints remain the greatest deterrent to the widespread use of pCLE. The initial expense of technology (approximately $150,000 for the equipment) plus the disposable probe expense ($500 to $800 per procedure) and increased procedural times of approximately 5–15 min pose challenges in the financially constrained healthcare systems [74]. Such costs are hard to justify in low-prevalence settings where the additional diagnostic benefit could not offset the expenditure. In contrast, other imaging modalities have quite lower expense profiles. Chromoendoscopy, for instance, carries minimal up-front capital investment and low per-procedure expense but increases biopsy volume and therefore downstream pathology expense [75]. Narrow-band imaging, an option included in much of today’s endoscopy hardware, involves no hardware cost but has reduced overall sensitivity for dysplasia in IBD and Barrett’s esophagus compared to pCLE [76]. Few cost-effective analyses of pCLE have been reported to date, and none have tried to factor in variability in healthcare system organization, payment schemes, or the availability of resources. More rigorous economic appraisal would require decision-analytic modeling founded on disease prevalence, yield of diagnosis, false-positive and false-negative results, cost of operating capital, life of probe, and potential downstream cost savings from reduced biopsies and improved cancer detection. Currently, there is no randomized trial comparing pCLE with chromoendoscopy or NBI with cost-effectiveness as the primary outcome. This will require multicenter trials that incorporate prospective micro-costing and health economic analysis into study design. These trials would measure not only procedural efficiency and diagnostic accuracy but also patient-centered outcomes and downstream healthcare utilization. This integrated approach would give the robust economic evidence required to drive strategic evidence-based implementation of pCLE into varied healthcare environments.

Lastly, broader adoption of pCLE technology is also anticipated beyond gastroenterology. Fields such as urology, neurosurgery, and dermatology, which rely on real-time tissue evaluation, are increasingly exploring pCLE for intraoperative diagnostics. In urology, pCLE may assist in evaluating bladder and prostate lesions, guiding resections with precision [77]. Neurosurgical applications include intraoperative brain tumor margin assessment, while dermatology can benefit from non-invasive skin cancer diagnostics [78, 79]. These applications require miniaturization of probes and the development of tissue-specific imaging protocols, but early reports suggest feasibility and potential for substantial clinical impact [80, 81].

## Conclusions

Probe-based confocal laser endomicroscopy is a powerful endoscopic imaging tool that enables real-time, high-resolution visualization of mucosal microstructures, enhancing diagnostic accuracy across various gastrointestinal diseases. pCLE improves dysplasia detection, guides targeted biopsies, and reduces reliance on random sampling, thereby optimizing procedural efficiency and clinical decision-making. Emerging data support its role in surveillance of Barrett’s esophagus, inflammatory bowel disease, gastric intestinal metaplasia, and indeterminate biliary strictures. Although pCLE offers strong diagnostic performance, its effectiveness remains operator-dependent, limited by superficial imaging depth, small field of view, and high equipment costs. Future advancements—including AI-assisted interpretation, molecular imaging, and expanded use in other specialties, along with standardization of training and further RCTs—are essential to maximize clinical adoption and cost-effective utilization.
